# Vorinostat Enhances Cytotoxicity of SN-38 and Temozolomide in Ewing Sarcoma Cells and Activates STAT3/AKT/MAPK Pathways

**DOI:** 10.1371/journal.pone.0142704

**Published:** 2015-11-16

**Authors:** Valerie B. Sampson, Nancy S. Vetter, Davida F. Kamara, Anderson B. Collier, Renee C. Gresh, E. Anders Kolb

**Affiliations:** 1 Nemours Center for Cancer and Blood Disorders, Nemours/A.I. duPont Hospital for Children, Wilmington, Delaware, United States of America; 2 Department of Pediatrics, Division of Hematology and Oncology, Children's Healthcare of Mississippi, University of Mississippi Medical Center, Jackson, MS, United States of America; Johns Hopkins University, UNITED STATES

## Abstract

Histone deacetylase inhibitors (HDACi) have been evaluated in patients with Ewing sarcoma (EWS) but demonstrated limited activity. To better understand the potential for HDACi in EWS, we evaluated the combination of the HDACi vorinostat, with DNA damaging agents SN-38 (the active metabolite of irinotecan and topoisomerase 1 inhibitor) plus the alkylating agent temozolomide (ST). Drugs were evaluated in sequential and simultaneous combinations in two EWS cell lines. Results demonstrate that cell viability, DNA damage and reactive oxygen species (ROS) production are dependent on the sequence of drug administration. Enhanced cytotoxicity is exhibited *in vitro* in EWS cell lines treated with ST administered before vorinostat, which was modestly higher than concomitant treatment and superior to vorinostat administered before ST. Drug combinations downregulate cyclin D1 to induce G0/G1 arrest and promote apoptosis by cleavage of caspase-3 and PARP. When ST is administered before or concomitantly with vorinostat there is activation of STAT3, MAPK and the p53 pathway. In contrast, when vorinostat is administered before ST, there is DNA repair, increased AKT phosphorylation and reduced H2B acetylation. Inhibition of AKT using the small molecule inhibitor MK-2206 did not restore H2B acetylation. Combining ST with the dual ALK and IGF-1R inhibitor, AZD3463 simultaneously inhibited STAT3 and AKT to enhance the cytotoxic effects of ST and further reduce cell growth suggesting that STAT3 and AKT activation were in part mediated by ALK and IGF-1R signaling. In summary, potent antiproliferative and proapoptotic activity were demonstrated for ST induced DNA damage before or simultaneous with HDAC inhibition and cell death was mediated through the p53 pathway. These observations may aid in designing new protocols for treating pediatric patients with high-risk EWS.

## Introduction

Ewing Sarcoma (EWS) is the second most common primary bone malignancy in pediatric patients and accounts for approximately 200 of all new pediatric cancer cases per year [1]. Current standard of care for EWS is a 5-drug chemotherapy regimen consisting of neoadjuvant and adjuvant vincristine, doxorubicin, cyclophosphamide, ifosfamide and etoposide, with surgery and/or radiation [2]. Patients with localized disease have a long-term survival rate of approximately 75%. The 5-year survival among patients with metastatic disease remains less than 30% [3, 4], and there are no effective treatments for relapsed disease. Identification and development of novel approaches for EWS are needed to prolong survival in patients with relapsed or refractory disease.

The hallmark of EWS is the t(11;22) (q24;q12) translocation that most frequently results in the EWS-FLI1 aberrant chimeric gene fusion. The EWS-FLI1 chimeric transcription factor regulates genes involved in oncogenesis. Despite knowledge of the tumor-initiating event, developing effective molecular targeting strategies for the EWS-FLI1 protein remains a challenge [5]. Inhibition of insulin-like growth factor-1 receptor (IGF-1R) signaling and the mammalian target of rapamycin (mTOR) pathways have been investigated as targeted therapies in EWS [6, 7]. Dramatic responses have been reported in a few patients, but constitutive and acquired resistance is common [8], which suggests that strategies combining these agents with standard cytotoxic drugs are needed. In the development of new therapies, it is important to establish the efficacy and mechanism of tumor cell death when drug combinations are used.

Epigenetic regulation of gene expression by histone deacetylases (HDAC) is an important event in oncogenesis. Vorinostat (suberoylanilidehydroxamic acid, SAHA), is an oral HDAC inhibitor (HDACi) that promotes apoptotic cell death in transgenic mouse models of medulloblastoma [9]. The initial testing of vorinostat by the pediatric preclinical testing program (PPTP) demonstrated no objective responses for solid tumors or acute lymphoblastic leukemia xenografts [10]. These results suggest that HDAC inhibition alone is not sufficient to treat pediatric cancers and that further evaluation of vorinostat in drug combinations is required to determine whether it has therapeutic value against pediatric cancers. The ability of HDACi to modulate transcription and gene expression has been shown to increase the cytotoxicity of other drugs in several cancer types [11, 12].

Temozolomide is an alkylating agent that methylates DNA primarily at O6-guanine, and shows single-agent activity (50–60%) in glioblastoma multiforme [13], melanoma [14] and anaplastic astrocytoma [15]. A COG Phase II trial of temozolomide in children with relapsed brain tumors demonstrated complete or partial responses in 4 of 25 patients with medulloblastoma/primitive neuroectodermal tumor (PNET) [16]. Irinotecan is a semisynthetic analogue of the natural alkaloid camptothecin that inhibits topoisomerase 1 and prevents DNA unwinding. It is used in the treatment of colon cancer, in combination with 5-fluorouracil and leucovorin [17]. Combining temozolomide and irinotecan was tolerable and active in EWS patients with advanced disease [18, 19]. The phase I testing of vorinostat in combination with temozolomide in recurrent or refractory brain or spinal cord tumors [16] was also well-tolerated in children. Three patients exhibited stable disease and one patient had a partial response. However, *in vitro*, upregulation of the DNA repair enzyme O(6)-methylguanine-DNA methyltransferase (MGMT) can repair DNA damage to confer drug resistance [16]. Further resistance to DNA damage may be mediated by activation of intracellular signaling pathways that inhibit cytotoxic signals in cancer cells. These events allow tumor cells to repair DNA damage and resume cell proliferation. The tumor suppressor p53 serves an important function as a cell cycle checkpoint protein. p53 regulates cell fate in response to toxic stimuli by triggering DNA repair or the apoptotic machinery [20]. In addition, in EWS the phosphatidylinositol-3 kinase/AKT (PI3K/AKT) and mitogen-activated protein kinase (MAPK) pathways have been shown to protect tumor cells from apoptosis and promote drug resistance [21, 22]. Consequently, understanding how mechanisms governing disease pathogenesis and molecular events are affected by agents is critical in the evaluation of therapies under development for this disease.

Studies determining the efficacy of combining HDACi and cytotoxic drugs demonstrated optimal sequence-specific combination strategies in breast and lung cancer [11, 12] but not in glioblastoma [23]. This study evaluated molecular mechanisms underlying the interactions of vorinostat and SN-38 (the active metabolite of irinotecan) plus temozolomide in preclinical models of EWS. We analyzed drug activity in three dosing combinations and determined the effects on DNA damage, cell-cycle, cell proliferation, and apoptosis. In addition, we evaluated vorinostat, SN-38 and temozolomide with the AZD3463 compound, which inhibits AKT and STAT3 pathways downstream of ALK and IGF-1R. A clearer understanding of the interactions between cytotoxic drugs and HDAC inhibition will be informative for the development of rational combination therapies for pediatric EWS patients.

## Materials and Methods

### Materials

Antibodies against histone H2A.X (H2AX), γ-H2AX (S139), ATM, phospho-ATM (S1981), cyclin D1, cleaved PARP, caspase-3, p53, phospho-p53 (S15), acetyl-p53 (K382), p44/42 mitogen-activated protein kinase (MAPK), phospho-p44/42 MAPK (T202/Y204), AKT, phospho-AKT (S473), histone 2B (H2B), acetyl-H2B (K5), histone deacetylase 1 (HDAC1), O-6-methylguanine-DNA methyltransferase (MGMT), signal transducer and activator of transcription 3 (STAT3), phospho-STAT3 (Y705), anaplastic lymphoma kinase (ALK), phospho-ALK (Y1604), insulin-like growth factor receptor (IGF-1R), phospho-IGF-1R (Y1132), phospho-Src kinase (Y416) and GAPDH were purchased from Cell Signaling (Danvers, MA). SN-38 (S), Temozolomide (T), Vorinostat (V), MK-2206 and AZD3463 were from Selleck Chemicals (Houston, TX). Stock solutions of compounds were prepared in DMSO and stored at -20°C or diluted in media for experiments.

### Cell lines and cell cultures

The human EWS cell lines, A4573 and TC32 were kindly provided by Jeff Toretsky, MD (Georgetown Lombardi Comprehensive Cancer Center, Washington D.C.) [24]. Cells were grown in RPMI 1640 medium (Invitrogen, CA) and supplemented with 10% fetal bovine serum (ATCC) containing 2 mM L-glutamine, 25 U/mL penicillin, and 25 μg/mL streptomycin (Invitrogen, CA). Cells were maintained in 37°C incubators, in an atmosphere of 5% CO_2_ with 100% humidity.

### Cell Proliferation Assay

To measure viable cells, A4573 and TC32 cells seeded in 96-well tissue culture plates (2 x 10^3^ cells/well) in medium without 1% antibiotics, were grown for 24 h then subjected to treatments at drug concentrations of 0.2 ng/ml SN-38 (S), 95 μg/ml temozolomide (T), 200 ng/ml vorinostat (V). SN-38 and temozolomide were administered together as a single agent (ST). Six experimental groups consisting of untreated cells (C), cells receiving ST (ST) and vorinostat (V) alone, and cells treated with agents in three combinations of ST followed by vorinostat alone (ST/V), vorinostat followed by ST alone (V/ST), and given simultaneously (STV), as outlined in Table 1. For single agent treatments (ST or V) and STV combination, cells received drug-free media for 24 h, followed by respective treatment for 24 h. For sequence combinations, cells received one agent for 24 h, which was removed by washing with phosphate-buffered saline (PBS), then incubated with the next agent for 24 h. Viable cells were measured at 48 h using the 3-(4,5-Dimethylthiazol-2-yl)-2,5-diphenyltetrazolium bromide (MTT) assay. 50 mg/mL MTT reagent was added, and incubated at 37°C for 4 h. The resulting formazan crystals were dissolved in DMSO (Fisher Scientific, PA). Absorbance values were read at 570nm using a Victor4 plate reader (Perkin Elmer, Waltham, MA). Cell viability assays were also performed with or without 5.0 μM MK-2206 and 20 nM AZD3463. Data is represented as the mean of six independent measurements ± SE.

**Table 1:** SN-38 plus temolomide and vorinostat treatment groups.

SN-38 (S): 0.2 ng/ml; Temozolomide (T): 95 μg/ml; Vorinostat (V): 200 ng/ml; Media (M)						
	Untreated	Single treatments		Combination treatments		
**Time**	C	*ST	V	ST/V	V/ST	STV
**24h**	M	M	M	ST	V	M
**24h**	M	ST	V	V	ST	STV

*ST: SN-38 plus Temozolomide administered together as a single agent

### Detection of reactive oxygen species (ROS)

A4573 and TC32 cells were seeded into black poly-lysine-coated 96-well plates at 2 x 10^3^ cells/well and grown for 24 h in serum-free medium (Corning, Union City, CA). Cells were subjected to treatments (Table 1). After 48 h 10 μM, dichlorofluorescein diacetate (DCFA) (Sigma, St. Louis, MO) was added singly or together with the ROS scavenger 5 mM N-acetyl-cysteine (NAC) (Sigma, St. Louis, MO). Cells were incubated at 37°C and 5% CO_2_ for 30 min to permit dye uptake and hydrolyzation. Excess dye was removed by washing with PBS. Fluorescence emission was measured in a Victor4 plate reader (Perkin-Elmer, Waltham, MA) at an excitation of 480nm and emission of 535nm. Data represent the mean of three independent measurements ± SE.

### Comet Assay

TC32 cells were treated as indicated in Table 1 then analyzed using the Comet Assay kit (Trevigen, Inc.) following manufacturer’s protocol. Briefly, cells were mixed with LM Agarose at 1:10 (v/v), coated on a CometSlide then lysed at 4°C for 1 h. Slides were placed in a horizontal electrophoresis tank containing electrophoresis solution (0.3 M NaOH and 1 mM EDTA, pH>13), denatured for 30 min and electrophoresis conducted at 20 V for 40 min (1 V/cm). After, slides were rinsed with 0.4 M Tris, pH 7.5 neutralization buffer, washed in distilled water, immersed in 70% ethanol for 5 min and dried at 40°C for 10 min. Cells were stained with SYBR green 1 dye (Molecular probes, Invitrogen, CA) and images were captured using a Leica fluorescent microscope. Comets (five fields per slide; ∼50 cells) were counted at ×100 magnification; counts were normalized to background (0.1% DMSO). Images were analyzed using Image-J software. DNA damage was expressed as tail length (average length of the tails) and percentage of DNA in tail (the tail intensity relative to the total cell DNA intensity). Values are the mean ± SE of three independent measurements.

### Annexin-V/PI Double-Staining Apoptosis Assay

Cell flow cytometry analysis was conducted to measure apoptotic cells using an Annexin V-FITC Apoptosis Detection Kit (BD Biosciences, USA). A4573 and TC32 cells exposed to 48 h drug treatments (Table 1) were harvested by trypsinization, washed with PBS, pelleted, resuspended in binding buffer and subsequently incubated with annexin V-FITC and propidium iodide (PI) at 1 μg/ml. Stained cells were loaded on C6 Accuriflow cytometer (BD Biosciences) for FL1 (annexin V) and FL2 (PI) bivariate analysis. Data from 200,000 cells/sample were collected. The percentages of early (annexin V-FITC positive and propidium iodide negative) and late (annexin V-FITC positive and propidium iodide positive) apoptotic cells were calculated.

### Western blot

After 48 h drug treatments (Table 1), A4573 and TC32 cells were washed with PBS and lysed in radioimmunoprecipitation assay (RIPA) buffer (50 mmol/L Tris-HCl pH 7.5, 150 mmol/L NaCl, 1% Triton X-100, 0.1% SDS, and 1% sodium deoxycholate), containing protease and phosphatase inhibitors (Invitrogen, CA). Lysates were sonicated, clarified by centrifugation and protein concentrations measured using the BCA protein assay (Pierce, Rockford, IL, USA). Proteins were separated on 10% acrylamide SDS–PAGE gels, transferred to nitrocellulose membranes and incubated with primary antibodies, followed by horseradish peroxidise-conjugated secondary antibodies (Cell Signaling, Danvers, MA). Blots were developed using chemiluminescence detection reagents (GE Healthcare, PA). GAPDH antibody was the loading control.

### HDAC activity assay

A4573 and TC32 cells were treated for 48 h (Table 1), washed with PBS and lysed in RIPA buffer. HDAC activity was measured using the HDAC Colorimetric Detection Assay Kit (Upstate Cell Signaling Solutions, NY). HDAC assay buffer (2X) and 4 mM HDAC assay substrate were added to lysates and incubated at 37°C for 90 minutes followed by diluted activator solution. Absorbance was measured at 405nm (Victor4 plate reader, Perkin-Elmer, Waltham, MA). Data represents the mean of three independent measurements ±SE.

### Imaging

Phase-contrast images were obtained using a Nikon Eclipse 80i microscope equipped with a DS-5M camera and NIS-Elements F Package (Nikon Inc., NY).

### Statistical analysis

Experiments were performed at least three times and values expressed as mean ±SE. p-values were calculated using the standard t-test. Differences were statistically significant at 95% confidence, when p<0.01.

## Results

### 1. Cytotoxic effects of SN-38 and Temozolomide (ST) combined with Vorinostat (V) on EWS cell lines

**Fig 1:**
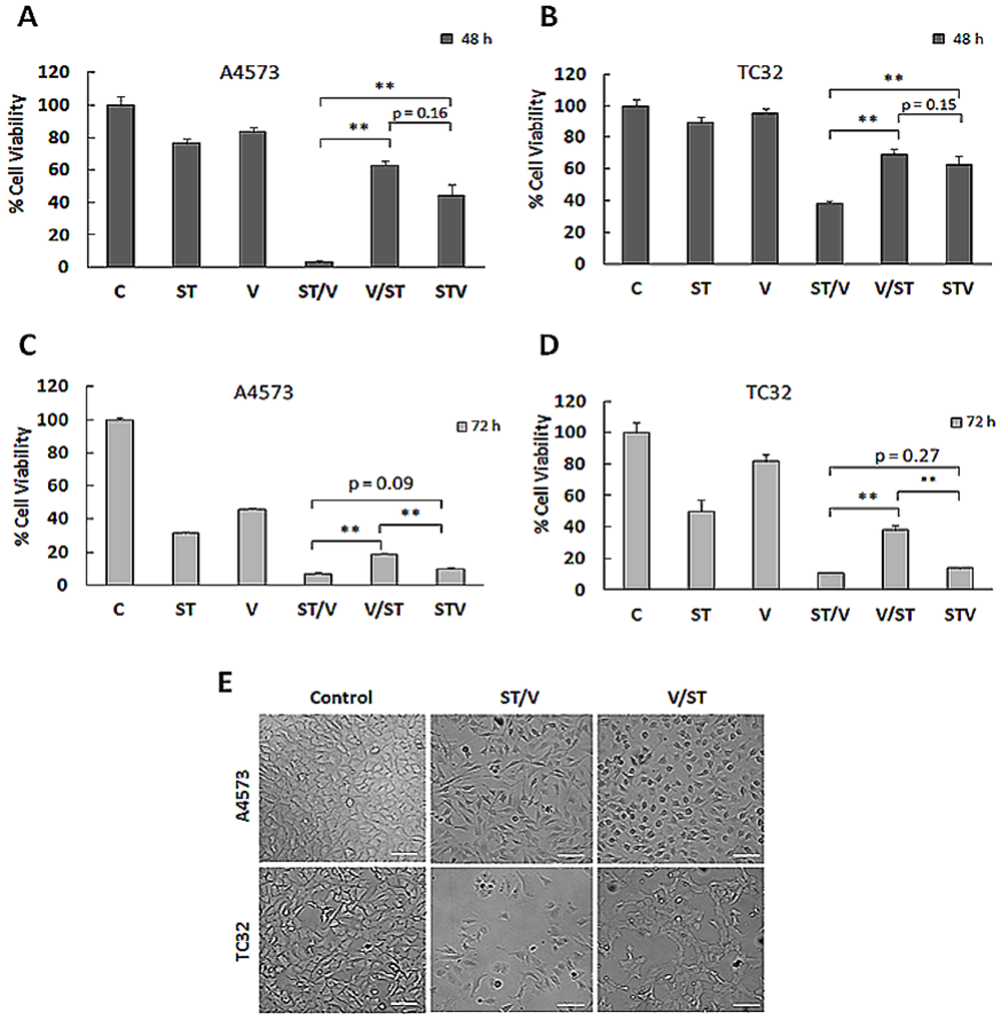
Effects of combining SN-38 plus Temozolomide (ST) and Vorinostat (V) on EWS cell proliferation. **(A)** A4573 cells were treated with SN-38 plus temozolomide (ST) and vorinostat (V) as single agents; in sequence combinations of ST followed by vorinostat (ST/V), and vorinostat followed by ST (V/ST) and simultaneous combination (STV), detailed in Table 1. For single agents alone and STV combination, cells received drug-free media for 24 h, followed by respective drugs for 24 h. For sequence combinations, cells received a single agent alone for 24 h, followed by the other agent alone for 24 h. Viable cells were measured at 48 h using the MTT assay. Data are presented as mean absorbance ±SE of six replicates, n = 6. Asterisks denote statistically significant differences for drug combinations, **p< 0.01. **(B)** Percentage of viable TC32 cells following 48 h drug treatments. **(C)** After 48 h single agent and combination treatments, cells received drug-free media for an additional 24 h. Cell viability was measured at 72 h by the MTT assay. **(D)** Percentage of viable TC32 cells subjected to 48 h drug treatments followed by 24 h incubation with drug-free media. **(E)** Phase contrast images depicting morphology of TC32 control cells and ST/V and V/ST treatment groups. Scale bar-200 μm.

The antiproliferative effects of topoisomerase I inhibitor SN-38, alkylating agent temozolomide and HDAC inhibitor vorinostat were first determined on two EWS cell lines, A4573 and TC32 using the MTT assay. The IC_50_ values were 0.2 ng/ml for SN-38, 95 μg/ml for temozolomide and 200 ng/ml for vorinostat following 72 h treatments (S1 Fig). Next, the effects of three different dosing combinations of ST and vorinostat (V) were evaluated on EWS cell growth. Drugs were administered sequentially, ST followed by vorinostat alone (ST/V), vorinostat followed by ST alone (V/ST), and simultaneously (STV), as summarized in Table 1. To achieve low toxicity of single agents, the incubation period for individual agents was 24 h. Fig 1A shows that after exposure to ST and vorinostat alone, viable A4573 cells were 76.2% (p<0.01) and 83.3% (p = 0.032), respectively, compared to untreated cells. After 48 h single agent treatments, when drugs were removed and cells incubated in drug-free media for an additional 24 h, cell proliferation further decreased (Fig 1C). This indicated that antiproliferative effects were sustained following drug removal. Effects of individual treatments on TC32 cells were less pronounced, but also showed increased efficacy over time (Fig 1B and 1D). When the three drugs were combined, there was greater cytotoxic activity on both cell lines. Specifically, for cells pretreated with ST before vorinostat (ST/V), the number of viable cells was significantly less than cells receiving vorinostat before ST (V/ST), (Fig 1A and 1B). This difference was maintained after drug removal and cells were incubated in drug-free media for an additional 24 h (Fig 1C and 1D). The STV simultaneous treatment with all three agents was as effective as the ST/V sequential treatment at 24 h after the last treatment. These experiments show that antiproliferative effects continued after drugs were removed, and also demonstrate that the differences in cytotoxicity for the sequential treatments were not merely a function of time.

Notably, distinct differences in cell morphology were observed following exposure to ST/V, V/ST and STV. As shown in Fig 1E, cells exposed to ST/V and STV (not shown) were spindle-shaped with long processes, while V/ST treated cells demonstrated morphology similar to controls. These morphological changes are suggestive of early responses to apoptotic stimuli in the ST/V and STV treatment groups [25]. Therefore, the cytotoxic effects of ST and vorinostat are sequence-specific in EWS cells.

### 2. ST and vorinostat induce DNA damage and ROS generation in EWS cell lines

**Fig 2:**
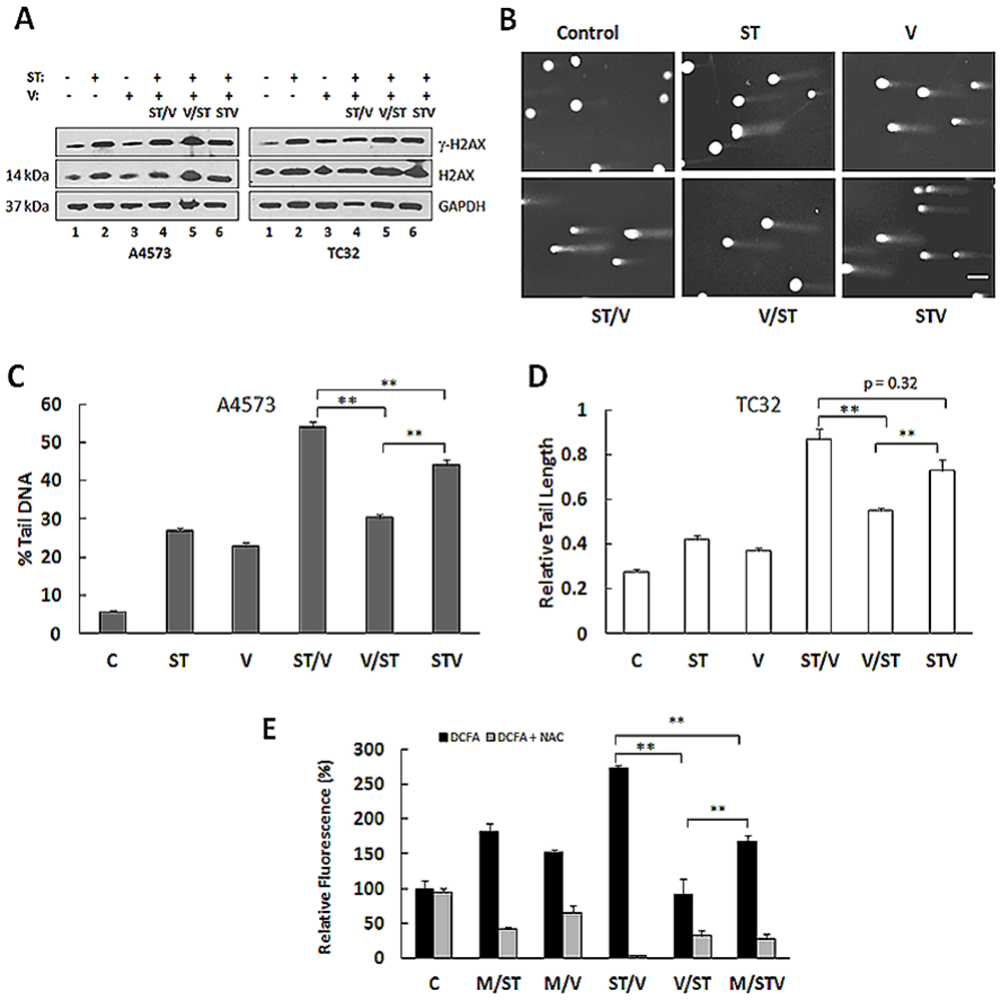
DNA damage and intracellular ROS generation in EWS cells following exposure to ST and vorinostat. **(A)** Immunoblot analysis of γ-H2AX in lysates of A4573 and TC32 subjected to single agents (ST and V) and drug combination (ST/V, V/ST and STV) treatments. GAPDH was loading control. **(B)** Comet images illustrating DNA fragmentation in TC32 cells treated with single agents and drug combinations. Comet assays were performed in triplicate. **(C)** The tail intensity relative to the total cell DNA intensity is represented as the percent tail DNA for each treatment group. Data shown represent mean ± SE for each experimental group (n = 50 cells). **(D)** The tail lengths of comets were measured in individual cells and expressed relative to the control group. Data shown represent mean ± SE for each experimental group (n = 50 cells). Asterisks denote significant differences for drug combinations, **p< 0.01. Asterisks denote significant differences in drug combinations, **p< 0.01 **(E)** Intracellular ROS production in TC32 cells following single and combination treatments was determined using dichlorofluorescein acetate (DCFA) dye. ROS fluorescence was quenched by N-acetyl-cysteine (NAC). Emitted fluorescence (Em/Ex 490/530 nm) in treated cells was compared with untreated control cells. Measurements were taken for 2 independent experiments, and the data are mean ± SE. Asterisks denote significant differences for drug combinations, **p< 0.01.

ST induces DNA damage [temozolomide promotes DNA double-strand (ds) breaks and SN-38 prevents religation of DNA strands], whereas vorinostat acetylates histones and loosens chromatin. To study DNA damage in EWS cells in response to drug combination treatments, we evaluated the phosphorylation of histone H2AX in the position of S139 (γ-H2AX) by immunoblot, and we performed the comet assay. γ-H2AX formation occurs at sites of DNA ds breaks and marks DNA damage. Fig 2A shows γ-H2AX generation in all treatment groups relative to untreated cells, and levels were highest for ST alone and the drug combinations. Consistent with these results, the comet assay showed largely condensed chromatin in untreated cells, while DNA fragmentation, depicted by comets were evident in the treated groups (Fig 2B). The proportion of cells with tail DNA increased following exposure to ST and vorinostat alone (Fig 2C), but the tails were not significantly longer than those of control cells (Fig 2D). Importantly, the percentage tail DNA and tail lengths were even greater with STV and ST/V treatments, whereas the effect was less pronounced in the V/ST group.

Cytotoxic agents and HDACis also generate ROS which could augment cellular injury. Total intracellular ROS accumulation in TC32 cells treated with ST, vorinostat and combinations was quantified using dichlorofluorescein diacetate (DCFA), a cell-permeable dye that is hydrolyzed by cellular esterases and emits fluorescence in response to oxidation. Fluorescence emission is shown in Fig 2E. Compared to controls, fluorescence increased following ST exposure (181 ± 12%, p<0.01) but to a lesser extent for vorinostat (151 ± 4.7%, p<0.01). Fluorescence levels were highest in cells receiving ST/V and STV treatments. Measurement for the ST/V group was 1.67 ± 0.08 fold-higher than the STV group (p<0.01) and 2.75 ± 0.2 fold-higher than the V/ST group (p = 0.32). Co-treatment with NAC effectively quenched intracellular ROS production and suppressed DCFA fluorescence in the treatment groups (Fig 2E). These results suggest that ROS production rapidly increases in response to drug treatments and likely contributes to cellular damage to enhance cytotoxicity of drug combinations. Taken together, DNA damage and ROS emission were enhanced when HDAC inhibition was concomitant with (STV), or followed by DNA damaging agents (ST/V).

### 3. ST and vorinostat promote cell cycle arrest and apoptosis in EWS cells

**Fig 3:**
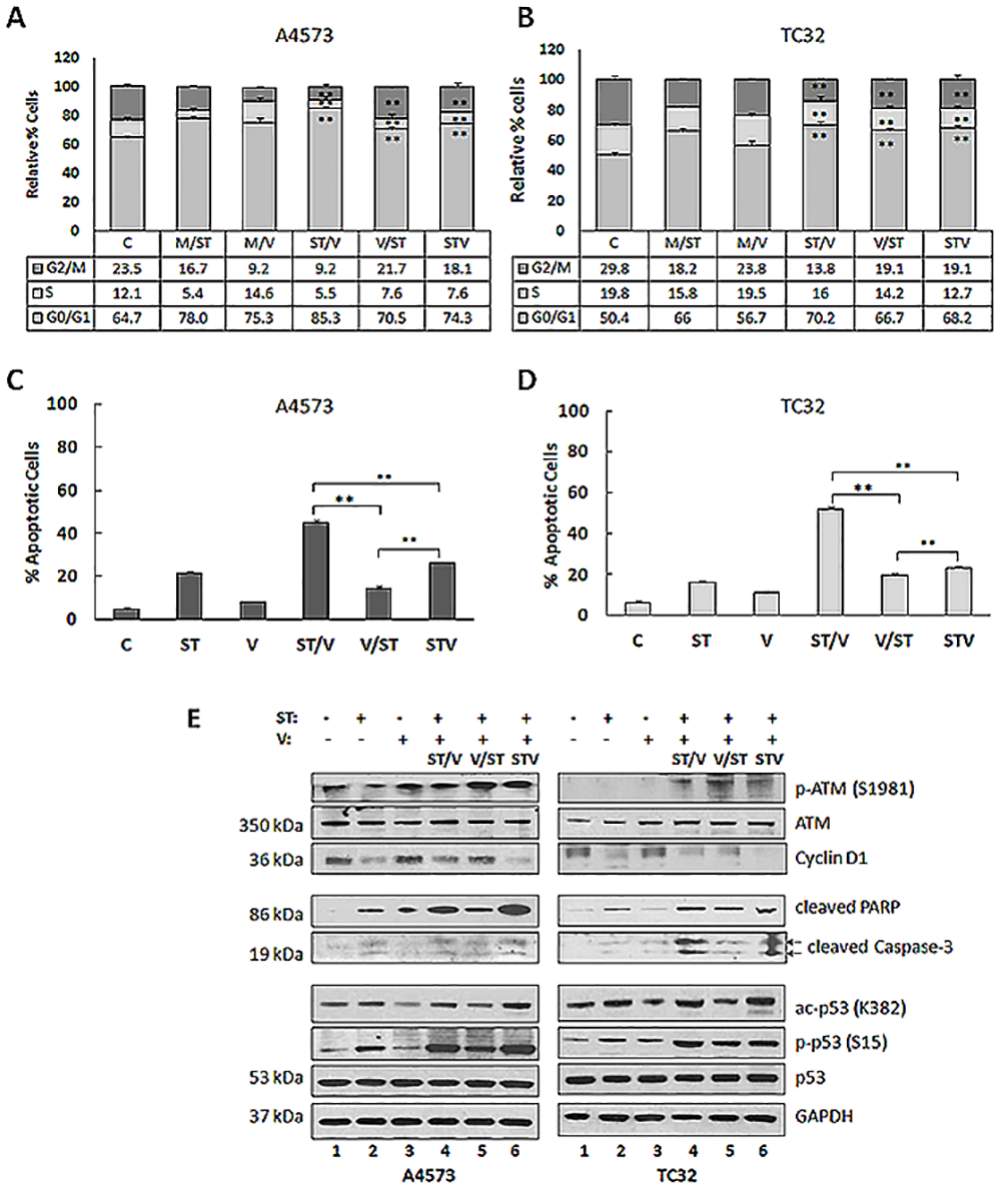
Cell cycle arrest and apoptosis in EWS cells following exposure to ST and vorinostat. **(A)** Control and treated A4573 cells were stained with propidium iodide (PI) and cell cycle analysis was conducted using flow cytometry. The percent distribution of cells in G0/G1, S and G2/M phase are depicted as histograms and values are listed in table format. Two individual experiments were performed and representative results are shown. **(B)** The percent distribution of TC32 cells in G0/G1, S and G2/M phase following single agent and drug combination treatments. **(C)** Plot representing A4573 cells undergoing late apoptosis (FITC-annexin V+/PI+) for all treatment groups. Data are presented as the mean ± SE (n = 3). Asterisks denote significant differences for drug combinations, **p< 0.01. **(D)** Plot representing TC32 cells undergoing late apoptosis (FITC-annexin V+/PI+) for all treatment groups. **(D)** Immunoblot analysis of A4573 and TC32 cell lysates following exposure to single (ST and V) and combination (ST/V, V/ST and STV) drug treatments, using antibodies against ATM, p-ATM (S1981), cyclin D1, cleaved PARP, cleaved caspase-3, p53, phospho-p53 (S15), and ac-p53 (K382). GAPDH was loading control.

With DNA damage, cell cycle arrest may occur to permit DNA repair or apoptosis. Cell cycle distribution was measured following drug treatments by propidium iodide (PI) staining of DNA and flow cytometry analysis. Fig 3A and 3B show that relative to controls, exposure of A4573 and TC32 cell lines to ST, ST/V, V/ST and STV resulted in the accumulation of cells in G0/G1 phase and a reduction of cells in G2/M. These results indicate G0/G1 phase cell cycle arrest and correlate with the expected cellular response to DNA damage. Double-strand breaks that occur during G1 phase can be retained at the G0/G1 checkpoint and repaired through non-homologous end joining [26]. Apoptosis was analyzed by double staining with PI and annexin V-FITC. In comparison to controls, the percentage of cells undergoing apoptosis was highest for the ST/V group (47.8% for A4573, 51.4% for TC32, p<0.01), and lower for the STV (27.8% for A4563, 22.6% for TC32, p<0.01), V/ST (14.8% for A4573, 18.6% for TC32, p<0.01), ST (21.1% for A4573, 14.9% for TC32, p<0.01) and vorinostat (7.8% for A4573, 9.9% for TC32, p<0.01) treatments (Fig 3C and 3D). Expression of checkpoint proteins that regulate the cell cycle, cyclin D1 (CD1), which regulates G1/S phase progression, and ataxia telangiectasia mutated (ATM) protein, which repairs DNA ds breaks [26] was assessed. Immunoblot analysis showed the decrease of CD1 protein after exposure to ST and drug combinations. Comparable reduction in cyclin D1 levels was observed for both sequence combinations, illustrating that G1 phase progression was blocked. Auto-phosphorylation of ATM on S1981 was observed in combination groups (Fig 3E) to further indicate initiation of the DNA damage response in cells [26]. Increases of the apoptotic markers cleaved PARP and cleaved caspase-3 were most prominent for ST, ST/V and STV treatment groups, in comparison to controls in both cell lines (Fig 3E). These results suggest that DNA damage induced by drug combinations effectively leads to cell cycle arrest to activate both DNA repair and cell death pathways. High DNA damage with the ST/V sequence facilitates apoptosis to eliminate damaged cells while lower DNA damage with the V/ST sequence facilitates the subsequent repair of DNA ds breaks to resume cell proliferation.

An important event in the cellular response to DNA damage is activation of the p53 protein which regulates transcription of genes promoting cell cycle arrest and apoptosis [20]. *In vitro* studies demonstrating that temozolomide-induced DNA damage response activates the ATM kinase and subsequent phosphorylation of p53 on Ser15 have been reported in several types of cancer, including human B lymphoblastoid cell line [27], melanoma cells [28] and glioma cells [29]. EWS cells and tumors predominantly express wild type-p53 [30], thus novel strategies that utilize the p53 pathway could enhance tumor cell death. Notably, wild-type and functional p53 have been demonstrated for several EWS cell lines [31–33] including TC32 [31, 34], but non-functional p53 is also reported for A4573 and TC32 cell lines [35]. p53 protein can be phosphorylated by ATM, ATR (ATM- and RAD3-related), and DNA-PK (DNA-dependent protein kinase catalytic subunit) at Ser15. Immunoblot analysis confirmed activation of p53 following exposure of A4573 and TC32 cells to ST, ST/V and STV treatments, by increases in acetylation and Ser15 phosphorylation (Fig 3D). Ac-p53 and p-p53 levels were lower for vorinostat and V/ST treated cells. Collectively, these results suggest that differential activation of p53 may provide protection against high DNA damage and ROS stress induced by rendering cells more susceptible to apoptosis in the ST, ST/V and STV treatment groups.

### 4. AKT, MAPK and STAT3 pathways are activated by ST and vorinostat treatments in EWS cells

**Fig 4:**
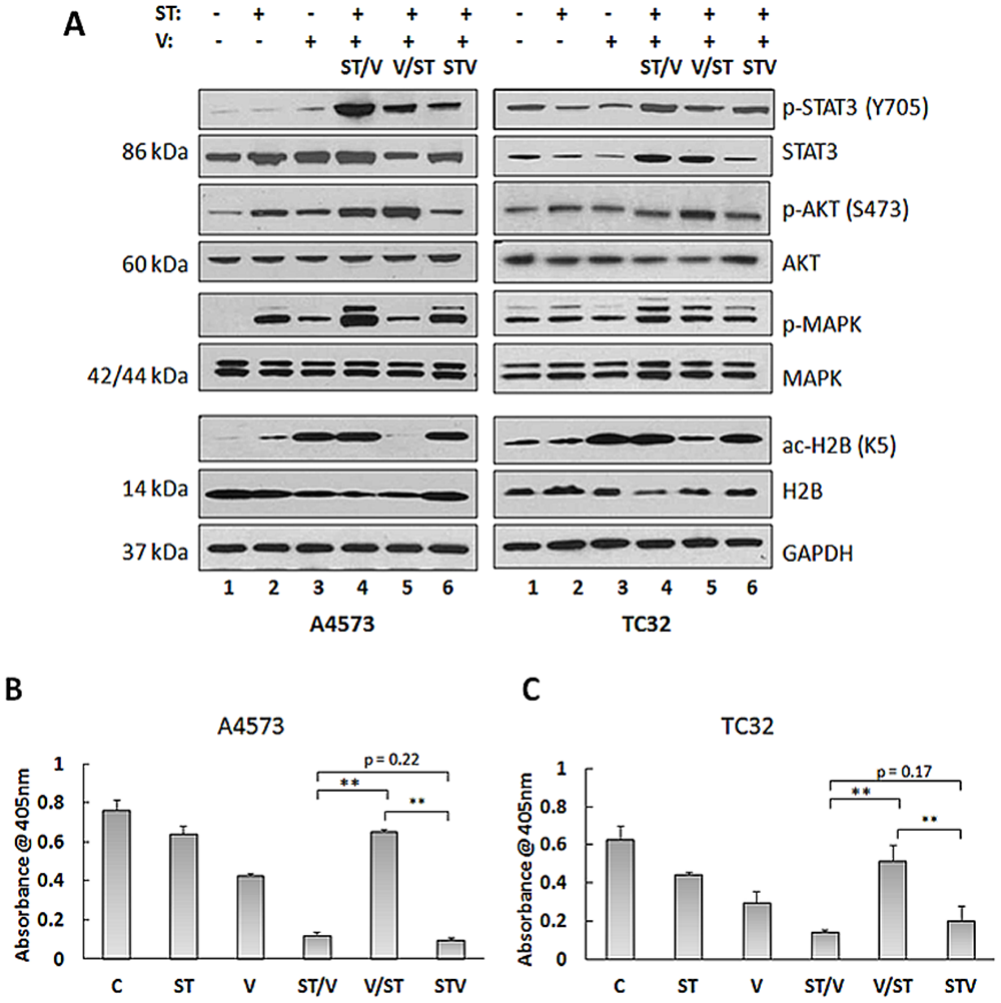
STAT3, AKT and MAPK activation in EWS cell lines following exposure to ST and vorinostat. **(A)** Immunoblot analysis of lysates of A4573 and TC32 cells following exposure to single (ST and V) and combination (ST/V, V/ST and STV) drug treatments, using antibodies against STAT3, p-STAT3 (Y705), AKT, p-AKT (S473), MAPK, p-MAPK (p42/44), histone 2B (H2B), and ac-H2B (K5). GAPDH was loading control. **(B)** HDAC activity was measured in A4573, and **(C)** TC32 cell lysates using the HDAC Activity Assay Kit (see Methods). Data are presented as mean absorbance (±SE), n = 3. Asterisks denote significant differences for drug combinations, **p< 0.01.

To further investigate the mechanisms regulating the effects of combining DNA damaging agents with HDACis on EWS cells, we examined the activation of major oncogenic signaling pathways, including AKT, MAPK and STAT3. PI3K/AKT signaling is critical for regulation of growth and tumorigenesis in EWS [21] and may also contribute to drug resistance [22]. Immunoblot analysis confirmed enhanced p-AKT levels in A4573 and TC32 cells following exposure to single and combination treatments, relative to controls (Fig 4A). Notably, highest p-AKT levels were observed for V/ST treatments. In addition, p-MAPK levels also increased in A4573 treated cells, and to a lesser extent in TC32 cells. In contrast to p-AKT levels, MAPK phosphorylation was highest for the ST/V group. Fig 4A also shows upregulation of p-STAT3 in both cell lines. In a similar manner to p-MAPK, phosphorylation was highest in the ST/V treatment group. Src and JAK family tyrosine kinases cooperate to mediate STAT3 activation. JAK protein was not detected by immunoblot in untreated or treated A4573 and TC32 cell lines (data not shown). In contrast, there was constitutive p-Src expression in A4573 cells (S2 Fig), and phosphorylation levels were reduced for TC32 cells receiving drug combinations, relative to controls. Our data suggests that Src may have a critical role in the activation of STAT3 that may involve tyrosine kinase signaling pathways. These results demonstrate that treatments also activated key pathways responsible for cellular proliferation, as a secondary drug response.

HDAC target inhibition was next assessed using anti-acetylated H2B antibody (ac-H2B, K5). Fig 4A shows accumulation of ac-H2B in cells that were treated with vorinostat, ST/V and STV. Unexpectedly, ac-H2B was reduced in V/ST treated cells (Fig 4A) and for other core histones (H2A, H3 and H4, data not shown). The effects of individual agents on acetylation status were further characterized in cells pretreated with vorinostat for 24 h followed by SN-38 alone, temozolomide alone, or drug-free media alone for 24 h (V/S, V/T or V/M, respectively). Ac-H2B was induced after 24 h exposure to vorinostat, and was present after an additional 24 h incubation period in drug-free media. When vorinostat pretreatment was followed by SN-38 alone (V/S) or temozolomide alone (V/T), accumulation of ac-H2B was lower (S3 Fig). To investigate these observations, total HDAC activity in both cell lines was measured using a colorimetric assay. As expected, HDAC activity was inhibited by vorinostat and also by ST/V and STV treatments compared to controls, Fig 4B and 4C. Notably, HDAC activity was high for the V/ST sequence, which is consistent with the reversal of H2B acetylation following V/ST treatments, Fig 4A. HDACis are thought to “prime” cells for DNA damage through histone acetylation and subsequent loosening of DNA. Our data demonstrates that for V/ST, when HDAC inhibition is replaced by DNA damage, HDAC inhibition is rapidly reversed and histone acetylation is reversed. This reactivation of HDACs diminishes the cytotoxic effect induced by vorinostat when followed by ST. Thus, augmentation of the DNA damaging effects of ST required continued HDAC inhibition concurrent to or following DNA damage, and not as a priming effect. Together these data demonstrate that the V/ST sequence provides EWS cells with compensatory survival capabilities mediated through AKT activation and decreases in histone acetylation.

### 5. AKT activation and MGMT upregulation diminish sensitivity of EWS cells to ST and V drug combinations, and concomitant AKT/MAPK/STAT3 inhibition enhances cell death

**Fig 5:**
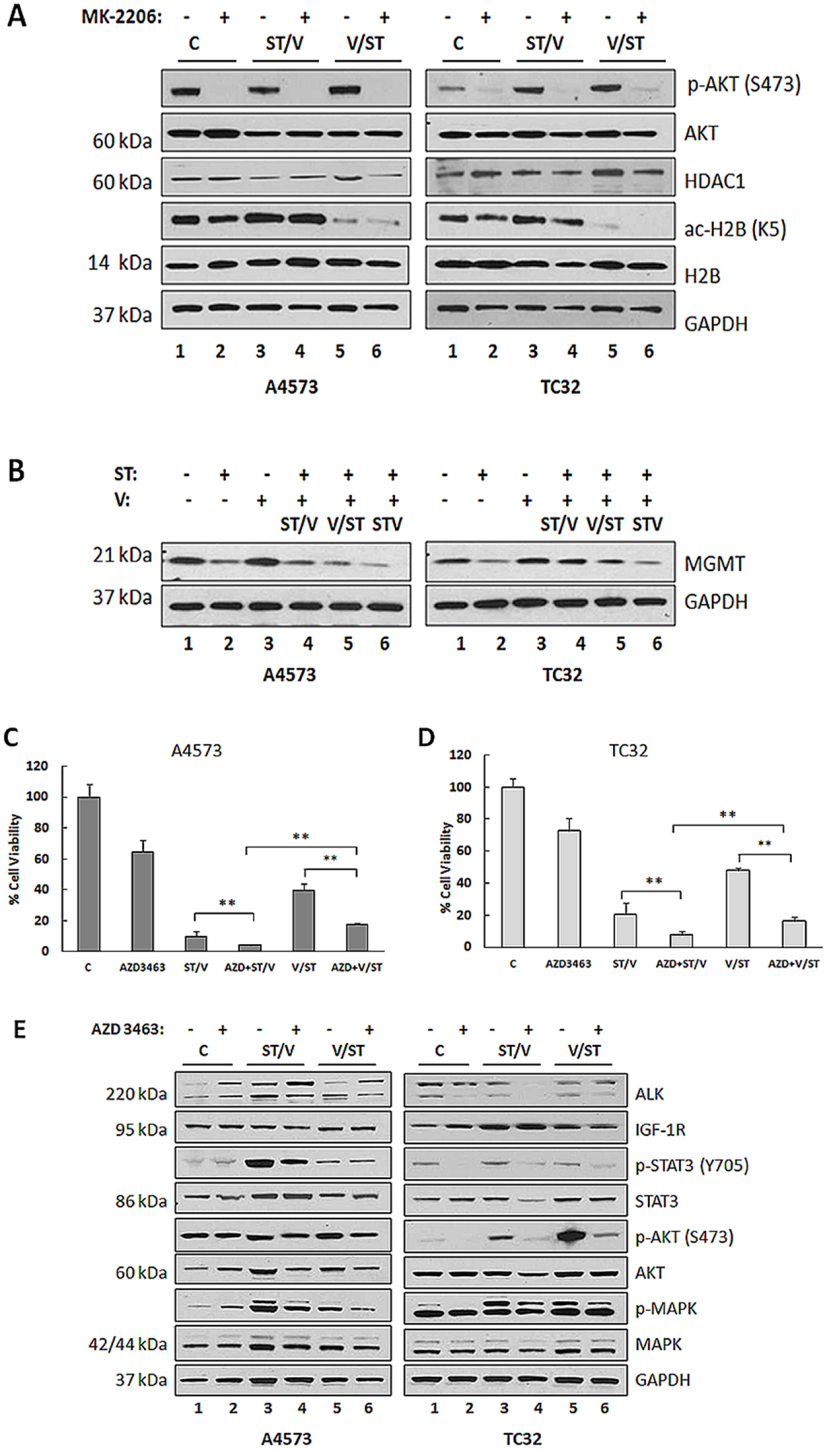
STAT3, AKT and MAPK inhibition in EWS cell lines following exposure to ST and vorinostat, MK-2206 and AZD3463. **(A)** Immunoblot analysis of lysates of A4573 and TC32 cells following exposure to media only (Control, C); ST/V and V/ST with (+) or without (-) 5.0 μM MK-2206 using antibodies against AKT, p-AKT (S473), HDAC1, histone 2B (H2B) and ac-H2B (K5). GAPDH was loading control. **(B)** Immunoblot analysis of A4573 and TC32 cells following exposure to single (ST and V) and combination (ST/V, V/ST and STV) drug treatments, using an antibody against MGMT. **(C)** A4573 cells and **(D)** TC32 cells were incubated with media only (Control, C); ST/V and V/ST with (+) or without (-) 20 nM AZD3463 and viable cells were measured at 48 h by the MTT assay. Data represents mean absorbance (±SE), n = 6. Asterisks denote significant differences between (+) versus (-) 20 nM AZD3463, **p< 0.01 **(E)** Immunoblot analysis of lysates of A4573 and TC32 cells following exposure to media only (Control, C); ST/V and V/ST with (+) or without (-) 20 nM AZD3463 using antibodies against ALK, IGF-1R, STAT3 (Y705), p-STAT3, AKT, p-AKT (S473), MAPK, p-MAPK (p42/44).

Upregulation of AKT has been reported in resistance mechanisms to HDAC inhibition in renal cell carcinoma [36] with no correlation to HDAC expression or activity. To determine whether there was any association between AKT activation and HDAC inhibition, A4573 and TC32 cells were incubated with either ST/V or V/ST in the presence of AKT inhibitor, MK-2206. As shown in Fig 5A, immunoblot analysis confirmed that neither total HDAC expression nor activity was affected by AKT inhibition, as ac-H2B was observed in the presence and absence of p-AKT. Furthermore, reversal of H2B acetylation in the V/ST group was unaffected, further validating these were separate events. In addition to histone acetylation, HDAC inhibition includes chromatin remodeling and transcription initiation. Since the MGMT protein is involved in the repair of DNA damage induced by temozolomide and is associated with drug resistance [16], MGMT expression was assessed by immunoblot. Vorinostat lead to upregulation of MGMT protein levels but MGMT expression did not increase with ST, ST/V and STV treatments (Fig 5B). While treatment with vorinostat increased MGMT, when this was followed by ST, MGMT remained at low levels. This data shows induction of MGMT by HDACi, a known mechanism limiting its efficacy in glioblastoma xenografts [37], is opposed by ST treatment in EWS cells.

The activation of AKT, MAPK and STAT3 signaling pathway in EWS is known to occur downstream of several growth factor receptors including ALK and IGF-1R [22, 38]. Aberrant ALK and IGF-1R signaling have been demonstrated to contribute to oncogenic transformation in pediatric sarcomas [39] and the involvement of IGF-1R signaling in resistance to HDACi was reported in NSCLC [40]. To confirm whether AKT, MAPK and STAT3 activation was mediated by ALK and IGF-1R, EWS cells were exposed to either ST/V or V/ST treatments in the presence of the dual ALK/IGF-1R inhibitor, AZD3463. The IC_50_ values for AZD3463 for A4573 and TC32 cell lines were 15 nM and 20 nM respectively, following 48 h treatments (S4 Fig). Target inhibition by AZD3463 is shown for cells treated with 20 nM compound, through reduction in p-ALK and p-IGF-1R levels (S5 Fig). Fig 5C and 5D show that blockage of ALK and IGF-1R signaling by ALK/IGF-1R inhibition sensitized A4573 and TC32 cells to ST/V and V/ST treatments through concomitant inhibition of p-MAPK and p-STAT3 (Fig 5E). While AZD3463 inhibits AKT activation in TC32 cells, AKT phosphorylation was unaffected in A4573 cells (Fig 5E). This is consistent with the observation that A4573 cells lack PTEN [41], and thus manifest constitutive AKT phosphorylation, not subject to regulation by these upstream receptors. It is possible that AZD3463 augments the effect of ST and vorinostat, in part by compromising DNA repair in EWS cells. These results indicate that ALK and IGF-1R activation may contribute to the development of resistance to HDACi and that dual targeting of ALK and IGF-1R is a potential strategy to prevent resistance to HDACi in EWS.

## Discussion

The strategy of combining DNA-damaging agents irinotecan plus temozolomide was effective in patients with relapsed EWS [19] and is now being tested in combination with targeted agents such as the aurora kinase A inhibitor MLN8247 (NCT01601535). In EWS xenografts, irinotecan and temozolomide in combination with PARP inhibitors achieved a response in more than 80% of mice [42]. These studies support the efficacy and safety of combining irinotecan and temozolomide with targeted agents. The HDACi vorinostat is clinically approved for the treatment of cutaneous T-cell lymphoma and has modest single-agent activity in advanced AML and myelodysplastic syndrome [43] and limited activity in EWS [10, 19]. The single-agent activity of HDACi in clinical trials has been generally low and improving the benefits of vorinostat is being explored in combination with cytotoxic agents. In this report, SN-38 and temozolomide were tested with vorinostat with a focus on identifying molecular patterns of drug response. Understanding mechanisms of action will allow the development of effective combination strategies to maximize the cytotoxic effect of this class of compounds in this disease.

The sequence-specific administration of vorinostat with SN-38, was suggested to potentiate drug effects in several cancer types including breast, lung, melanoma and glioblastoma [11, 12, 23, 44]. However, there is no consensus on optimal strategies in pediatric patients. In the current study, combining SN-38 with temozolomide and vorinostat achieved favorable toxicity in EWS cell lines. Furthermore, using different dosing schedules, DNA damage and cellular responses were induced to varying extents, demonstrating that drug efficacy is sequence-dependent. ST given before vorinostat and concomitantly, demonstrated high cytotoxicity *in vitro*, and was superior to the administration of vorinostat given before ST. Mechanistically, the induction of DNA strand breaks with ST, followed by or concurrent with HDAC inhibition (ST/V and STV respectively) renders cells more susceptible to DNA damage than when HDAC inhibition precedes DNA strand breaks (V/ST). Accordingly, the V/ST sequence was also the least toxic, although the combination did not appear antagonistic. The production of endogenous ROS was also proportional to DNA damage to generate oxidative stress that enhances the potency of drug treatments. These results suggest that the efficacy of DNA damaging agents with HDAC inhibition lies in the balance of DNA damaging signals and that cytotoxicity is proportional to the magnitude of DNA damage.

The ability for cells to repair DNA lesions to maintain genomic integrity represents another important determinant of treatment efficacy. Activation of DNA repair pathways can reverse the cytotoxicity of DNA-damaging agents providing a key mechanism of cancer cell resistance. DNA damage decreased cyclin D1 protein, and increased PARP and capase-3 cleavage to promote G0/G1 cell cycle arrest and activation of proapoptotic events. Importantly, activation of the base excision repair pathway was demonstrated through phosphorylation of ATM protein. ATM is a protein kinase that is recruited and activated by DNA ds breaks to initiate DNA repair and phosphorylates several target proteins including the p53 tumor suppressor. Moreover, continued histone acetylation also inhibits DNA repair by activation of the p53 pathway. Several mechanisms are thought to influence p53 tumor suppressor inactivation in EWS cell lines including EWS-FLI1 mediated silencing [45] and degradation of p53 by Mdm2 [32]. p53 and MAPK activation are associated with mechanisms controlling cellular events in response to DNA damage, including p53 phosphorylation [46, 47], p38MAPK interaction [48] and activity of proteins involved in apoptosis [49]. While *TP53* is not considered a clinically relevant biomarker in EWS [50], these studies show that EWS cells can exploit the global changes of cytotoxic chemotherapy and HDAC inhibition to activate p53.

**Fig 6:**
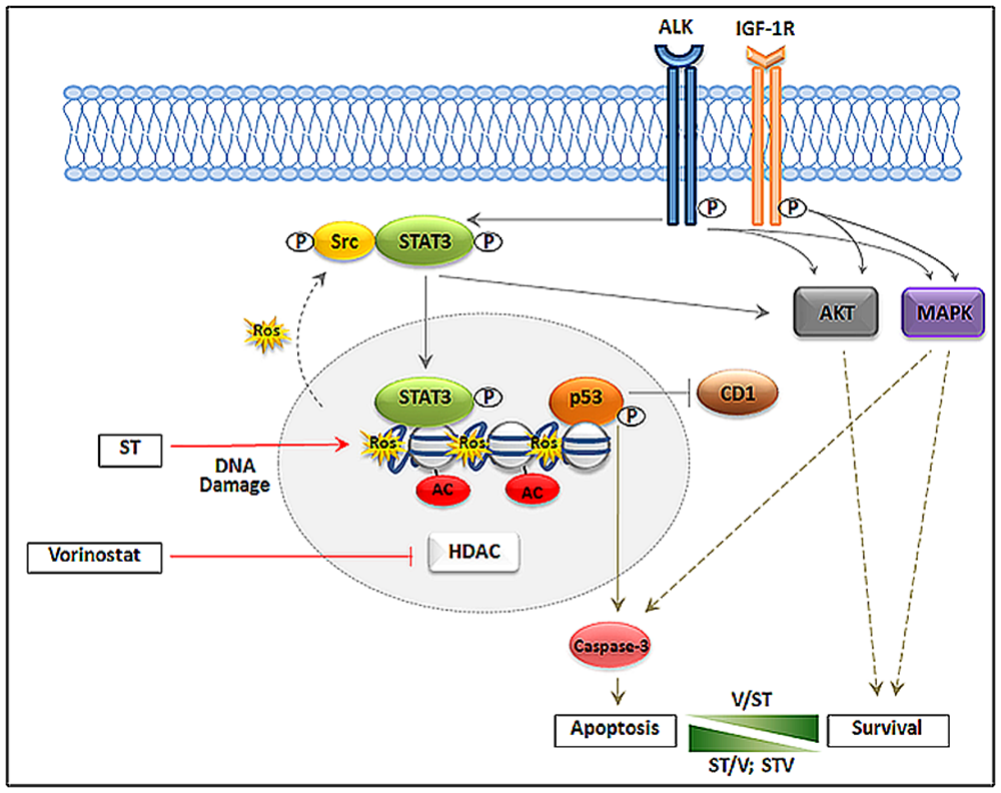
Summary of EWS cellular response to ST and vorinostat drug combinations. The figure depicts EWS cellular response following DNA damage (DNA double-strand breaks and ROS production) and HDAC inhibition. ST promotes DNA damage and vorinostat inhibits HDACs leading to activation of p53, inhibition of CD1, cleavage of caspase-3 and induction of apoptosis. A secondary drug response involves activation of STAT3, mediated by Src, and activation of AKT and MAPK mediated in part through ALK and IGF-1R. V/ST combination facilitates greater cell proliferation (Survival) and ST/V and STV combinations facilitate greater cell death (Apoptosis). Abbreviations: HDAC—Histone deacetylase; ROS—reactive oxygen species, CD1 –cyclin D1, STAT3 –signal transducer and activator of transcription 3; MAPK—mitogen-activated protein kinase, ALK—anaplastic lymphoma kinase; IGF-1R –insulin-like growth factor 1 receptor. Red arrows represent drug inhibition; Solid gray arrows represent constitutive signaling pathways; Solid blunt lines represent inhibition of signals; Dotted gray lines represent activation of signaling pathways in response to drug treatments.

The intracellular events promoting EWS cell survival are well-studied and signaling through AKT, MAPK and STAT3 are known to regulate cell proliferation, survival, differentiation and motility [21]. One therapeutic strategy that has been investigated is specific targeting of receptor tyrosine kinases (RTKs) implicated in these pathways. However, clinical benefits to this approach have been modest, except for a small subset of patients, mostly in relapsed EWS who achieved dramatic clinical response to monoclonal antibodies directed against IGF-1R. Further, work by our lab [22], along with others, have confirmed tumors can activate salvage signaling pathways to maintain cell growth and limit the clinical efficacy of these inhibitors. Other studies have also shown that inverse responses in activation between the AKT and MAPK pathways can influence apoptosis [51]. Furthermore, induction of STAT3 protein and activation by ST and vorinostat drug combinations is indicative of the cellular response to DNA damage. Thus, high p-STAT3 levels may be predictive of high DNA damage and sensitivity of tumors to drug treatments. These changes are depicted in Fig 6.

The current study demonstrates that activation of AKT in the V/ST treatment schedule may induce molecular changes that allow cells to evade the protective G1 arrest mediated by CD1 and attenuate cell death. Moreover, AKT activation may also protect cells from apoptosis induced by cytotoxic agents [22], since targeted inhibition of AKT by MK-2206 further enhanced cytotoxicity of drug combinations. This finding is consistent with previous reports that many standard and targeted therapies induce RTK activity to activate cell proliferation pathways and sustain cell survival [8]. Furthermore, cross-talk between receptors such as IGF-1R, insulin receptor (IR), epidermal growth factor receptor (EGFR), ALK and Met underlies a key mechanism in resistance to therapies involving several pathways including AKT, MAPK and STAT3. We have demonstrated that dual ALK/IGF-1R inhibition enhanced the sensitivity of EWS cells to ST and vorinostat through inhibition of p-AKT, p-MAPK and p-STAT3. This suggests that combining DNA damaging agents with HDACi and relevant RTK inhibition may simultaneously block the response to several survival signals that can activate compensatory survival responses in EWS. Accordingly, a comprehensive analysis of all key receptors is needed in order to develop a relevant targeted approach to AKT/MAPK/STAT3 signaling and is expected to increase the effectiveness of new therapies.

Pretreatment with vorinostat before ST (V/ST) reversed histone acetylation *in vitro* and was confirmed to be mediated through reactivation of HDACs. Furthermore, overexpression of the MGMT DNA repair protein following vorinostat treatments was implicated in reducing the efficacy of V/ST treatments. This important observation likely changes the early equilibrium between cell proliferating and cell death signaling pathways, to initiate signaling cascades that promote DNA repair and cell survival since MGMT expression is one of the primary mechanisms of resistance to temozolomide. Rationally designed combination therapies will expand the anticancer activity of conventional and targeted therapies, while countering mechanisms of constitutive and acquired drug resistance. To achieve this, a thorough evaluation of the molecular events that govern cellular responses to targeted and conventional therapies must be conducted in the preclinical assessment of new therapeutic strategies. In summary, we have identified several biologic consequences that are associated with both cell survival and cell death when combining SN-38, temozolomide and vorinostat in EWS. We suggest that combining these agents with the inhibition of AKT may enhance sensitivity of tumors to DNA damage and HDAC inhibition. Elucidation of these mechanisms may also identify biomarkers of sensitivity to support the use of rational combination therapies in this disease.

## Supporting Information

S1 Fig Effect of SN-38, temozolomide and vorinostat on viability of EWS cells. A4573 and TC32 cell lines were treated with different concentrations of SN-38, temozolomide and vorinostat for 72 h and cell viability was determined by the MTT assay. Plots show the percentage of viable cells compared to untreated cells. Data points represent mean absorbances ± SE of six replicates (±SE), n = 6 vehicle control.
(TIF)

S2 Fig Expression of p-Src in EWS cell lines treated with ST and vorinostat single agent and combination treatments. Immunoblot analysis of lysates of A4573 and TC32 cells following exposure to single (ST or V) and combination (ST/V, V/ST and STV) drug treatments, using an antibody against p-Src. GAPDH was loading control.
(TIF)

S3 Fig Histone acetylation accumulation in EWS cell lines treated with vorinostat followed by SN-38 or temozolomide as single agents. Immunoblot analysis of lysates of A4573 and TC32 cells following exposure to vorinostat followed by either drug-free media (V/M) or SN-38 (V/S) or temozolomide (V/T) for 24 h for 24 h using antibodies against H2B and ac-H2B (K5). GAPDH was loading control.
(TIF)

S4 Fig Effect of dual ALK/IGF-1R inhibitor AZD3463 on viability of EWS cells. A4573 and TC32 cells were treated with different concentrations of AZD3463 for 48 h and cell viability was determined by the MTT assay. Plots show the percentage of viable cells compared to untreated cells. Data points represent mean absorbances ±SE of six replicates (±SE), n = 6.
(TIF)

S5 Fig Target inhibition by dual ALK/IGF-1R inhibitor AZD3463 in EWS cell lines. Immunoblot analysis of lysates of A4573 and TC32 cells following exposure to AZD3463, using antibodies against ALK, p-ALK (Y1604), IGF-1R, p-IGF-1R (Y1132). GAPDH was loading control.
(TIF)
