# Supplementary figures and images for: Vorinostat Enhances Cytotoxicity of SN-38 and Temozolomide in Ewing Sarcoma Cells and Activates STAT3/AKT/MAPK Pathways
Source: PLoS One. 2015 Nov 16;10(11):e0142704. doi: 10.1371/journal.pone.0142704 (PMC4646493; doi:10.1371/journal.pone.0142704)

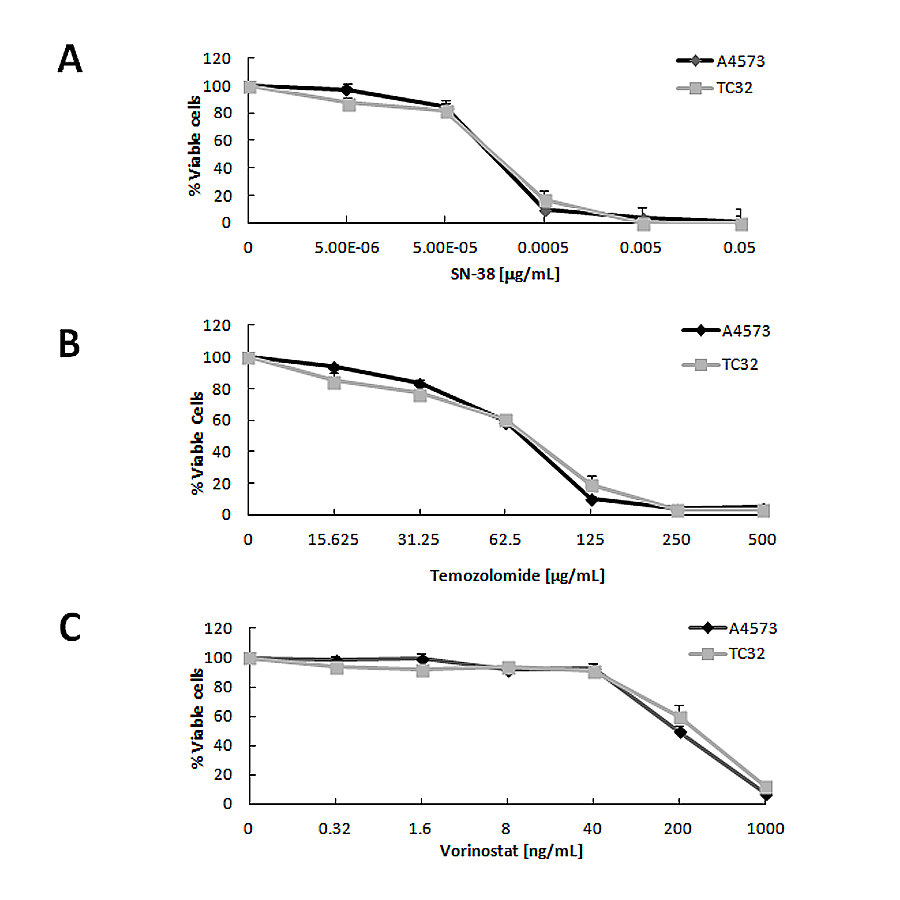

Supplement: S1 Fig — A4573 and TC32 cell lines were treated with different concentrations of SN-38, temozolomide and vorinostat for 72 h and cell viability was determined by the MTT assay. Plots show the percentage of viable cells compared to untreated cells. Data points represent mean absorbances ± SE of six replicates (±SE), n = 6 vehicle control. (TIF) [file pone.0142704.s001.tif]

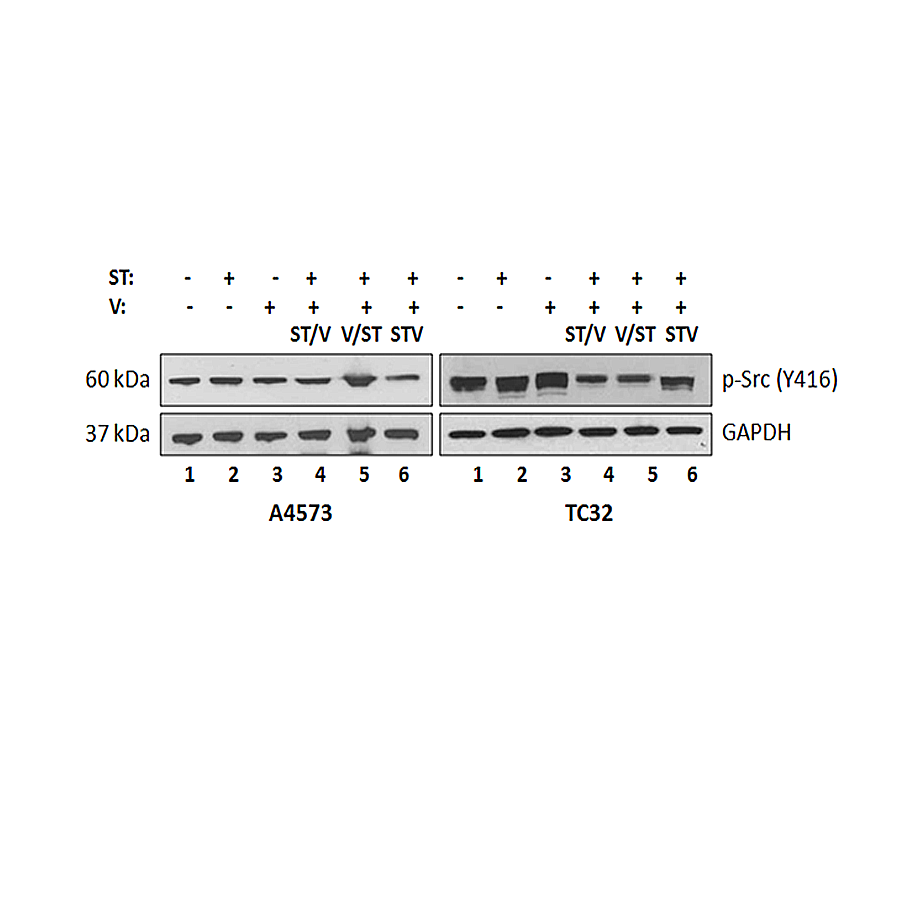

Supplement: S2 Fig — Immunoblot analysis of lysates of A4573 and TC32 cells following exposure to single (ST or V) and combination (ST/V, V/ST and STV) drug treatments, using an antibody against p-Src. GAPDH was loading control. (TIF) [file pone.0142704.s002.tif]

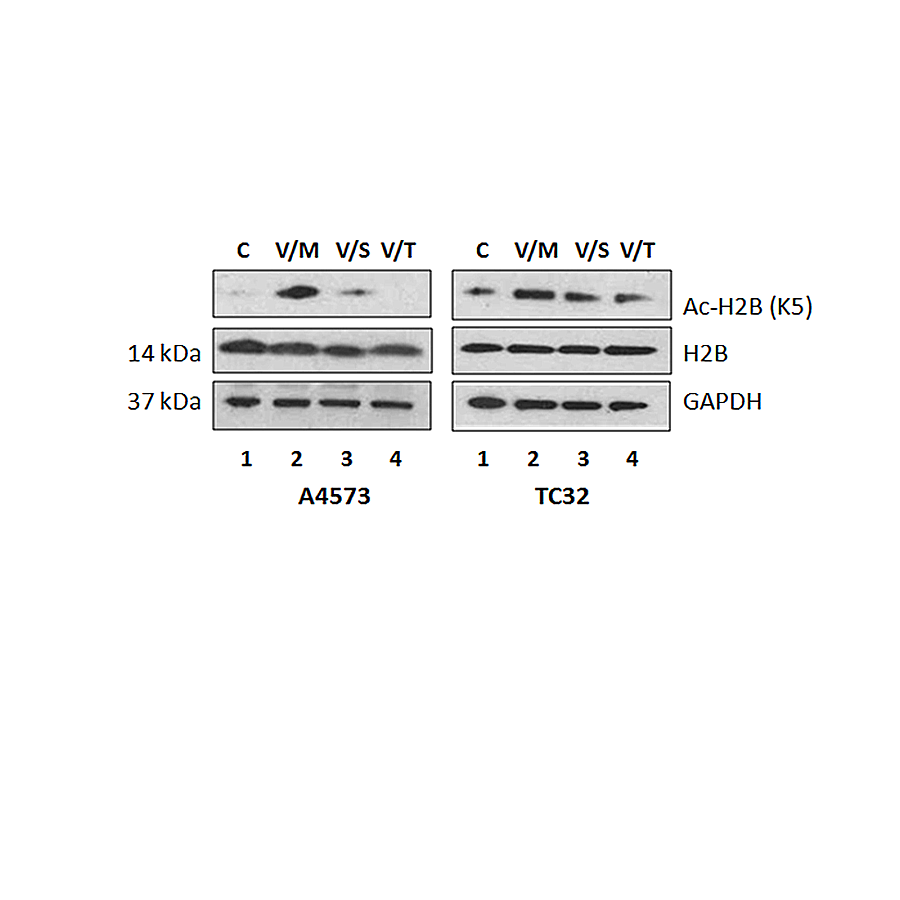

Supplement: S3 Fig — Immunoblot analysis of lysates of A4573 and TC32 cells following exposure to vorinostat followed by either drug-free media (V/M) or SN-38 (V/S) or temozolomide (V/T) for 24 h for 24 h using antibodies against H2B and ac-H2B (K5). GAPDH was loading control. (TIF) [file pone.0142704.s003.tif]

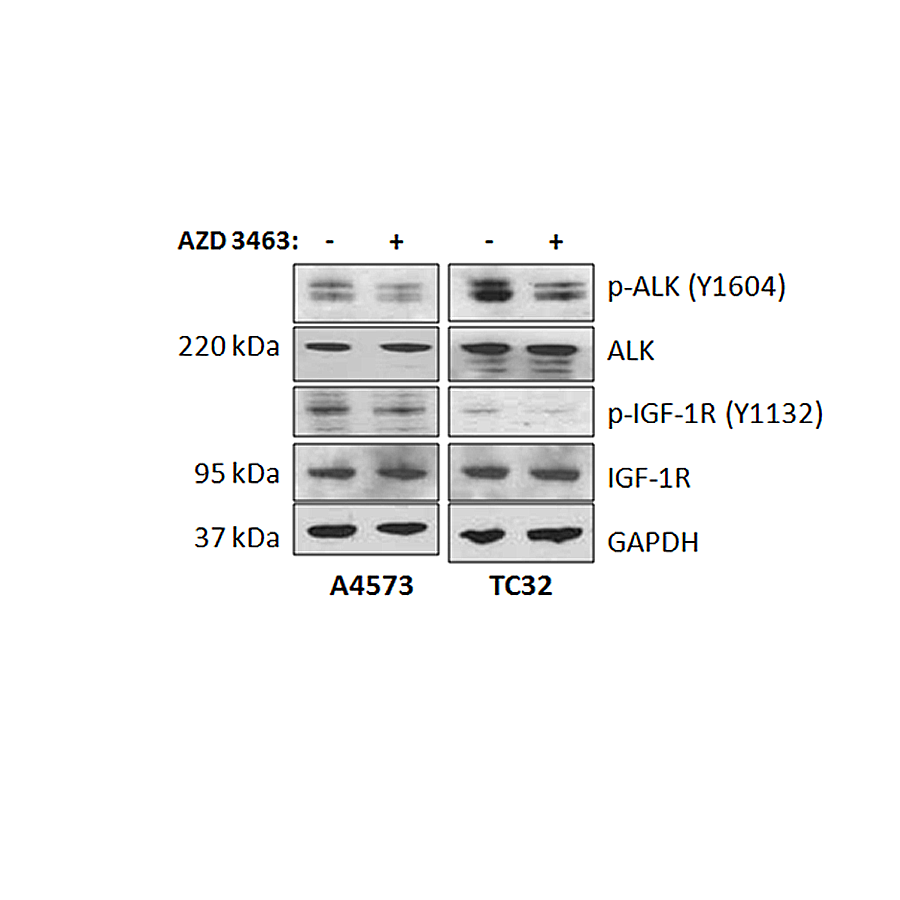

Supplement: S4 Fig — A4573 and TC32 cells were treated with different concentrations of AZD3463 for 48 h and cell viability was determined by the MTT assay. Plots show the percentage of viable cells compared to untreated cells. Data points represent mean absorbances ±SE of six replicates (±SE), n = 6. (TIF) [file pone.0142704.s004.tif]

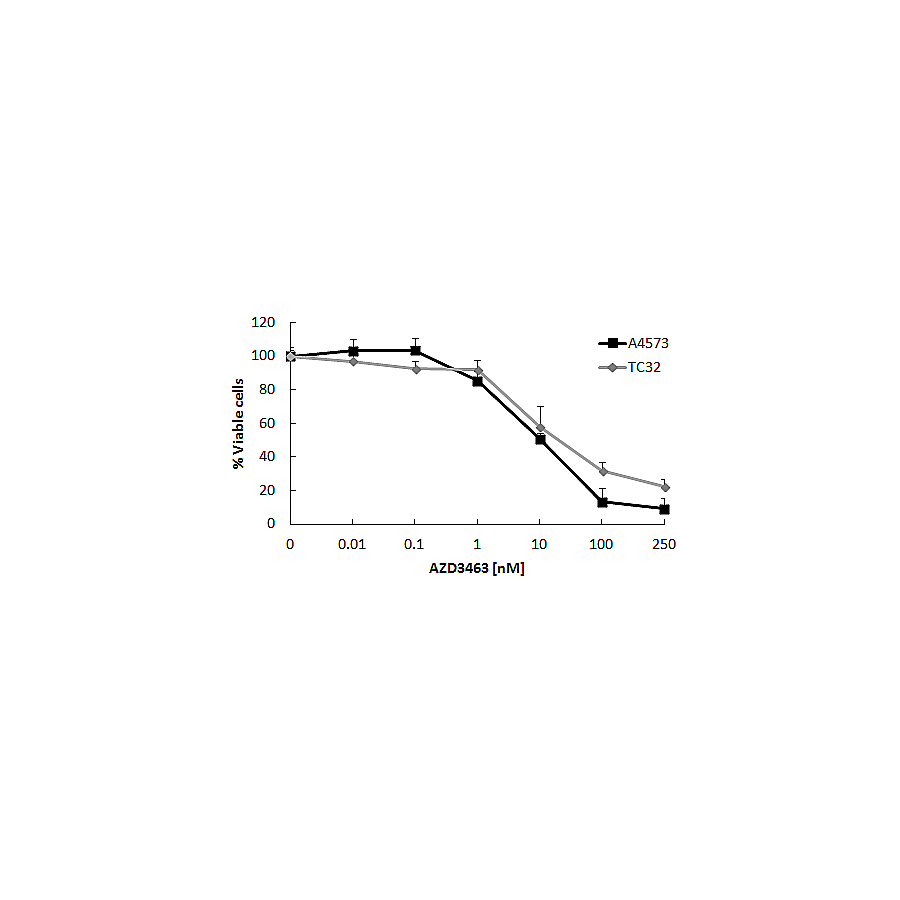

Supplement: S5 Fig — Immunoblot analysis of lysates of A4573 and TC32 cells following exposure to AZD3463, using antibodies against ALK, p-ALK (Y1604), IGF-1R, p-IGF-1R (Y1132). GAPDH was loading control. (TIF) [file pone.0142704.s005.tif]
